# Letter from Dr. R. P. Talley, of Belton, Referring to Report of Proceedings of Austin District Medical Society

**Published:** 1889-02

**Authors:** 


### CORRESPONDENCE.

### LETTER FROM DR. R. P. TALLEY, OF BELTON, REFERRING TO REPORT OF PROCEEDINGS OF AUSTIN DISTRICT MEDICAL SOCIETY.

"Dr. Talley's paper was read at the night session, but was not received and therefore not discussed. It was an attempted argument against medical legislation, but was in fact, no argument. It consisted mainly of personalities, ridicule and abuse of all former attempts at legislation by the organized profession."

*Editor Daniel's Texas Medical Journal*:

DEAR SIR:—The above, which is from the report of the fifth quarterly meeting of the Austin District Medical Society, published in the January, 1889, number of this MEDICAL JOURNAL over the signature of Dr. T. J. Bennett, Secretary, does me and my article, I think, a very grave and serious injustice. Had the article itself been published in the same issue of the MEDICAL JOURNAL, it would have been its own full and complete refutation of the above charges. But, as it was not, I deem it but just to myself and my professional brethren, who may not have seen my article, to correct the false impression about it that would be made by Dr. Bennett's report.

Since the publication of this report I have carefully re-read my article and I do not find any of the charges made in this report sustained by it. I did not make, or attempt to make, any argument against "medical legislation." I did say substantially that all attempts in the past, in this state, to *regulate* the practice of medicine, had proven failures. The reasons for these failures, I asserted, were two fold; first, the doctors themselves had never fully agreed upon a law for this purpose, and, second, that the people had not yet been educated up to supporting such a law. In this my position was but little, if any different from that taken by Dr. Wooten in his annual address before the society last September. (See *Daniel's Texas Medical Journal*, October, 1888, page 154). And his views seem to have met with the approbation of the society and of the JOURNAL.

I also advocated the established of a State Board of Health. I gave my reasons for this and in addition thereto said that this, if adopted, could be made the stepping stone to other medical legislation. In connection with argument in favor of a State Board of Health, I said that I thought all classes of physicians could agree upon it, and that it would be a good means of educating the people.

I did not "abuse all," or any "former attempts at legislation by the organized profession." I did criticise some of the laws passed by the legislature to regulate the practice of medicine, and I said those laws had proven failures. That this is true, is too patent to every physician to need substantiation. I also criticised some of the methods to secure medical legislation, which had been advocated by the editor of this journal, and some other physicians in the State, in their individual capacities. These methods had been advocated either by articles published in the medical journals, or by addresses delivered before some of the medical societies. They had become public property, and were the legitimate subjects of criticism. I did not say a word about any "attempts at legislation" that had ever been made by the "organized profession."

Upon a very careful examination of my article, I find the name of but one physician living in the State mentioned in it. That one was Dr. F. E. Daniel, the editor of this journal. I appeal to him if there is anything personal about him or any other physician, in the article. Personal, means "relating to, or affecting a person." Now, I submit in all candor and fairness, that in the entire article I did not use, directly or by innuendo, a word or sentence that, by any fair, legitimate construction of language, could be construed into a reflection upon the personal or professional character of any physician in the State.

I am more than willing that my article shall stand or fall upon its own merits, but I insist that it should not be judged by any misrepresentation of it, whether made intentionally or not. And, in this connection, I will say that I do not charge that Dr. Bennett has intentionally or wilfully misrepresented me, but the injustice to me would be just as great as if he had, if it were allowed to go without correction.

Respectfully submitted,

R. P. TALLEY.

---

Referring to the above letter, in justice to the writer and to ourselves, we will say that in publishing Dr. Bennett's report of the proceedings of the Austin District Medical Society as written, we had no thought or intent of doing Dr. Talley either injury or injustice; and while we do not disclaim responsibility for any article prepared by Dr. Bennett, who is kind enough to aid us materially in the labor of publishing the JOURNAL, and who has become identified with us in the capacity of collaborator,—we naturally supposed that readers would see that the report of the meeting and the language used expressed the views of the Secretary, and not of the JOURNAL.

We do not endorse Dr. Talley's paper. As it was understood and announced that the subject of Medical Legislation would be discussed at the meeting of the Austin District Medical Society referred to, the discussion to open upon a paper by Dr. Talley, which paper, it was understood, would be an argument against medical legislation, and so announced in the JOURNAL and by circular, we hold, and so expressed ourselves to Dr. Talley, that his paper was not a fair and full presentation of the subject of medical legislation from the standpoint of opposition. Hence the members of the Society who were present at the meeting (we were absent by reason of serious illness,) claim, and we believe it is what Dr. Bennett meant in his report, that the subject *not having been presented*, no discussion followed. Dr. Talley feels that the Society treated him badly. If they did, we regret it, but are in no way responsible for it. We did not intend to hurt him, for we have for him the kindest feelings, and had he contributed his paper to the JOURNAL instead of to the Galveston News, we would willingly have published it, for it is a very readable paper. It is an argument for the establishment of a Board of Health; which we take to be a very different thing from an argument vs. the regulation of the practice of medicine by statutory enactment, as is done in twenty-six States, in accordance with a decision of the courts that they have that right, in the interest of the public health.

As to personalities, we have not complained of anything offensive in that way in the Doctor's paper. Our name was used rather freely in connection with past efforts to secure medical legislation, bnt not in a way to be at all objectionable.

We publish the Doctor's letter as an act of justice to him, and trust that he may fully understand our position with reference to himself and to his paper.

